# Comparison of intrathecal morphine versus local infiltration analgesia for pain control in total knee and hip arthroplasty: A meta-analysis

**DOI:** 10.1097/MD.0000000000021971

**Published:** 2020-09-04

**Authors:** Bao-chang Qi, Jing Yu, Wei-song Qiao

**Affiliations:** a Department of Orthopedic Traumatology, the First Hospital of Jilin University, Changchun, Jilin, China; b Department of The First Operating Room, the First Hospital of Jilin University, Changchun, Jilin, China.

**Keywords:** local infiltration analgesia, meta-analysis, total hip arthroplasty, total knee arthroplasty

## Abstract

**Background:**

The purpose of this meta-analysis was to comprehensively collect randomized controlled trials (RCTs) to assess the clinical efficacy of intrathecal morphine (ITM) versus local infiltration analgesia (LIA) in the treatment of total knee and hip arthroplasty patients.

**Methods:**

Relevant studies were identified from the Embase, PubMed, Cochrane Library, Web of Science, Wanfang, and Chinese National Knowledge Infrastructure (CNKI) databases. We also reviewed the references of all identified articles to identify additional studies. For each study, we assessed the risk ratio (RR), weighted mean difference (WMD), and corresponding 95% confidence interval (95% CI) to synthesize outcomes. Meta-analysis was performed with Stata 12.0 software.

**Results:**

We included 13 studies with 942 patients for meta-analysis. LIA significantly decreased the pain value with rest or mobilization until 72 hours (*P* < .05). LIA significantly decreased cumulative morphine consumption by 13.52 mg. Moreover, the length of hospital stay was lower in the LIA group than in the ITM analgesia group. Finally, LIA significantly reduced morphine-related complications (nausea and vomiting, pruritus, and respiration depression).

**Conclusions:**

LIA was an effective approach for relieving postoperative pain and reducing postoperative consumption of morphine compared with ITM in total knee and hip arthroplasty patients.

## 1 Introduction

Total knee arthroplasty (TKA) and total hip arthroplasty (THA) are the most common procedures in orthopedic surgeries that can relieve joint pain and improve joint function.^[1–3]^ In the U.S., there are approximately 600,000 TKA procedures and 100,000 THA procedures performed each year. However, pain following TKA and THA in patients has been reported to be a common postoperative complication.

Careful pain management is necessary after TKA and THA to achieve early postoperative mobilization while ensuring patient comfort throughout.^[4]^ Moreover, effective pain control can quicken the recovery time and reduce the length of hospital stay.^[5,6]^

There are many analgesic options available for TKA and THA that vary in terms of efficacy and potential complications. Local infiltration analgesia (LIA) and intrathecal morphine (ITM) are 2 alternatives for pain management in TKA and THA patients. Some controversies remain, however, regarding the pain control efficacy and safety of these 2 methods. McCarthy et al^[7]^ conducted a randomized controlled trial and revealed that LIA conferred superior analgesia to ITM following TKA in patients. Hess et al^[8]^ also suggested that ITM may not have a morphine-saving effect and may increase the occurrence of pruritus at doses over 0.3 mg. However, different opinions exist. Kaczocha et al^[9]^ indicated that ITM reduces postoperative pain in TKA patients.

Therefore, it was necessary to conduct a systematic review and meta-analysis to compare the efficacy and clinical outcome of LIA and ITM in TKA and THA patients. We hypothesized that LIA was superior to ITM for pain control without increasing adverse events in TKA and THA patients.

## 2 Methods

The Cochrane Handbook for Systematic Reviews of Interventions was followed during the performance of this meta-analysis, and the full texts were written in accordance with the PRISMA checklist (Preferred Reporting Items for Systematic Reviews and Meta-analyses). Because the meta-analysis was based on aggregate data, ethical approval was not required.

### 2.1 Literature search

Two individuals independently reviewed the Embase, PubMed, Cochrane Library, Web of Science, Wanfang, and Chinese National Knowledge Infrastructure (CNKI) databases for relevant articles published through March 2020. We also reviewed the references of all identified articles to identify additional studies. The search terms were as follows: “local infiltration anesthesia,” “anesthesia, local,” “infiltration anesthesia,” “anesthesia, infiltration,” “local anesthesia,” “intrathecal morphine,” “intraspinal injections”; “injections, intraspinal,” “injection, intraspinal,” “intraspinal injection,” “spinal injections,” “injection, spinal,” “injections, intrathecal,” “total knee arthroplasty,” “total knee replacement,” “TKA,” “TKR,” “total hip arthroplasty,” “total hip replacement,” “THA,” “THR,” “random, randomized controlled trial,” and “RCT.” These key words and mesh terms were combined with “AND” or “OR.”

### 2.2 Inclusion and exclusion criteria

The PRISMA guidelines were followed for the inclusion of studies in the meta-analysis. Following the PICOS (Participants, Interventions, Comparisons, Outcomes and Study design) principle, the key search terms included (P) patients with total knee or hip arthroplasty; (I/C) patients treated by LIA or ITM; (O) the outcomes including the related clinical indexes [visual analog scale (VAS) with rest or mobilization until 72 hours, cumulative morphine consumption, length of hospital stay, occurrence of nausea and vomiting, pruritus, and respiration depression]; and (S) RCT.

The exclusion criteria included subjects in animal studies, cadaveric studies, in vitro studies, studies with a single cohort, case reports, and articles published in a form other than clinical trials. The article review procedure was conducted by 2 independent authors. If there was any disagreement between the 2 authors regarding the inclusion criteria of the related articles, a third author was consulted.

### 2.3 Data extraction

The data extraction was conducted by 2 independent authors separately by using a standardized form and recorded in Microsoft Excel (Microsoft Corporation, Redmond, WA). The following data were extracted: first author, publication year, Jadad score, intervention of LIA and ITM, sample size, age, female patients, and surgery type. The clinical outcomes included VAS with rest or mobilization until 72 hours, cumulative morphine consumption, length of hospital stay, occurrence of nausea and vomiting, pruritus, and respiration depression. Differences and disagreements were resolved by consensus. If the trials had more than 2 groups, we only extracted the interest-reported information and data. Pain intensity was assessed using the VAS pain score [range, 0 (no pain) to 10 (agonizing pain)].

### 2.4 Quality assessment

The quality of the included studies was assessed independently by 2 reviewers (Bao-chang Qi and Jing Yu) using the Jadad score, which evaluates studies based on randomization appropriateness, blinded outcome assessments, and complete descriptions of patients lost to follow-up.^[10]^ A Jadad score of 1 to 2 was considered low quality, and a Jadad score of 3 to 5 was considered high quality.

### 2.5 Statistical analysis

Meta-analysis was performed with the support of Stata 12.0 software (Stata Corp., College Station, TX). For dichotomous outcomes (the occurrence of nausea and vomiting, pruritus, and respiration depression), the results were calculated with risk ratios (RRs) and 95% confidence intervals (95% CIs). For continuous outcomes with normal distributions, weighted mean differences (WMDs) and 95% CIs were used to test the overall effects. Random- or fixed-effect models were adopted depending on the value of *I*^2^: when *I*^2^ > 50%, suggesting significant heterogeneity, a random-effect model was used in the pooled result; in contrast, a fixed-effect model was employed when *I*^2^ < 50%. Heterogeneity among the included studies was analyzed by the Chi-square test and *I*^2^. The *Z* test was used to evaluate the overall effect.

## 3 Results

### 3.1 Search results

**Figure 1:**
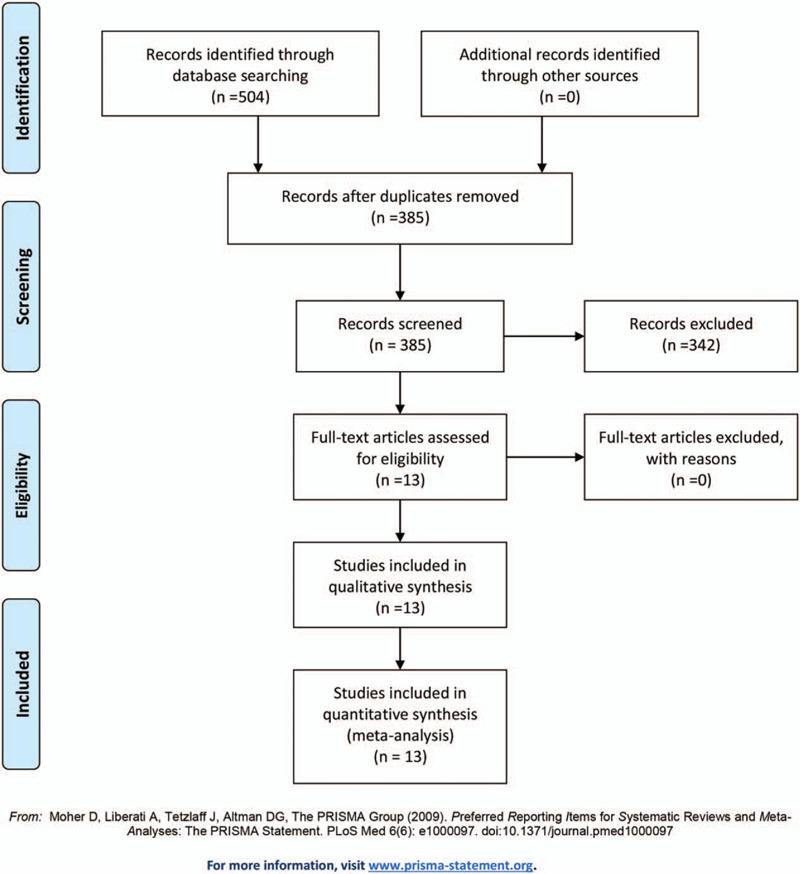
Flow chart of literature screening.

We reviewed a total of 504 articles identified by our initial keyword search, of which 119 were excluded following de-duplication by EndNote X7 (Thomson Reuters). Then, 342 studies were excluded after screening the title and/or abstract. After full-text reading and screening, the remaining 13 articles were retrieved and read, and no studies were excluded according to the inclusion criteria. Finally, we included a total of 13 studies^[7,11–22]^ for this meta-analysis, incorporating 942 patients. The study selection is outlined in Fig. 1.

### 3.2 Characteristics of the trials

Table 1 summarizes the basic information for each study, including author names, year of publication, interventions, sample, age, and sex. The main Jadad score was 3.73, indicating the high quality of the included studies. According to the intervention of the included studies, we divided them into 2 subgroups for analysis: TKA versus THA. LIA was mainly administered with the use of ropivacaine, bupivacaine, or levobupivacaine with or without epinephrine. The sample size in the LIA group ranged from 19 to 61, and that in the ITM group ranged from 15 to 50. The mean ages of the LIA and ITM groups were 66.9 and 66.8 years, respectively. A total of 8 studies compared LIA versus ITM for TKA, and the remaining 4 studies compared LIA versus ITM for THA.

**Table 1:**
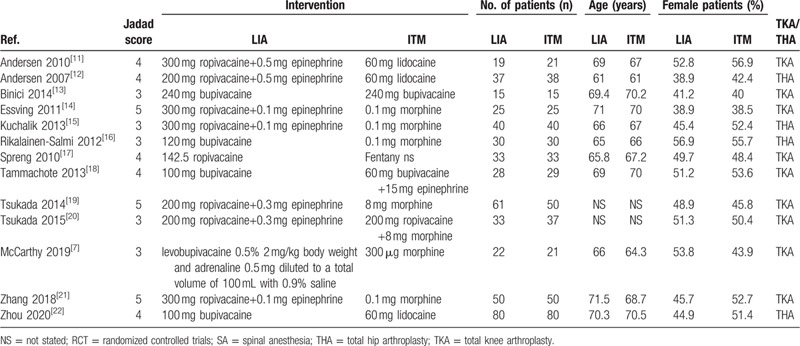
General characteristic of the included studies.

### 3.3 VAS with rest until 72 hours

The summary results of LIA versus ITM in terms of the VAS scores with rest up to 72 hours are summarized in Table 2. The pooled results indicated that LIA could significantly reduce the VAS score with rest at 6 hours (WMD: −7.12; 95% CI: −10.25 to −3.12; *P* = .000), 12 hours (WMD: −8.12; 95% CI: −12.25 to −2.52; *P* = .000), 24 hours (WMD: −6.78; 95% CI: −10.25 to −4.29; *P* = .000), 48 hours (WMD: −6.23; 95% CI: −11.52 to −1.06; *P* = .000), and 72 hours (WMD: −5.87; 95% CI: −9.13 to −2.69; *P* = .000).

**Table 2:**
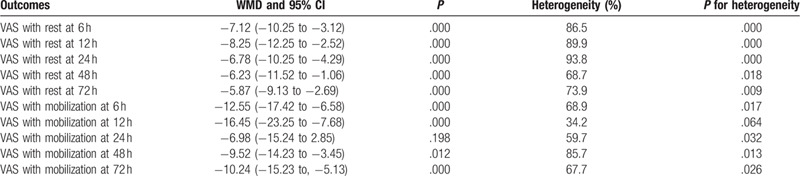
The summary results for VAS with rest or mobilization at 6, 12, 24, 48, and 72 h.

### 3.4 VAS with mobilization until 72 hours

The combined results showed a significantly lower pain score with mobilization in the LIA group at 6 hours (WMD = −12.55, 95% CI: −17.42 to −6.58, *P* = .000; Table 2), 12 hours (WMD = −16.45, 95% CI: −23.25 to -7.68, *P* = .000; Table 2), 24 hours (WMD = −6.98, 95% CI: −15.24 to 2.85, *P* = .198; Table 2), 48 hours (WMD = −9.52, 95% CI: −14.23 to −3.45, *P* = .012; Table 2), and 72 hours (WMD = −10.24, 95% CI: −15.23 to −5.13, *P* = .000; Table 2) than in the ITM group.

### 3.5 Total morphine consumption

**Figure 2:**
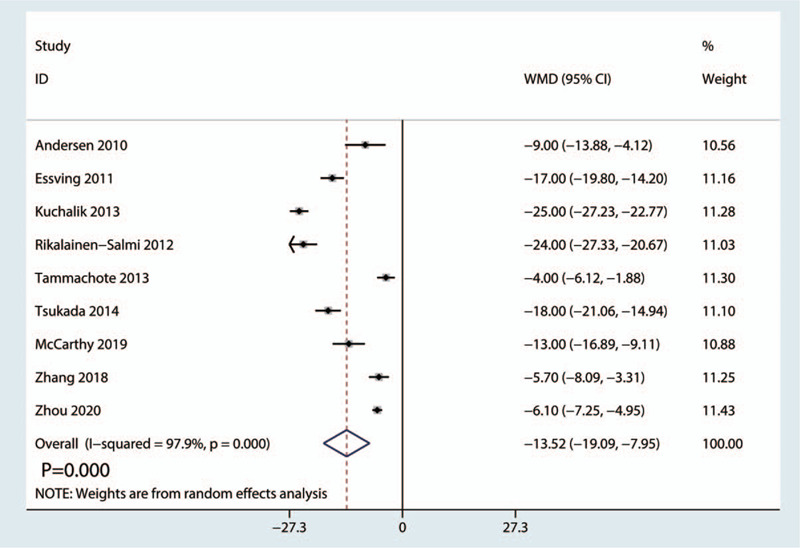
Forest plots of the included studies comparing the total morphine consumption between the 2 groups.

Nine trials totaling 659 patients (LIA = 335, ITM = 324) provided data on the total morphine consumption. The results showed that there was statistical heterogeneity in the total morphine consumption (*I*^2^ = 97.9%, *P* = .000, Fig. 2); the data were analyzed using the random-effects model.

LIA significantly decreased the total morphine consumption compared with ITM (WMD = −13.52, 95% CI: −19.09 to −7.95, *P* = .000; Fig. 2).

### 3.6 Length of hospital stay

**Figure 3:**
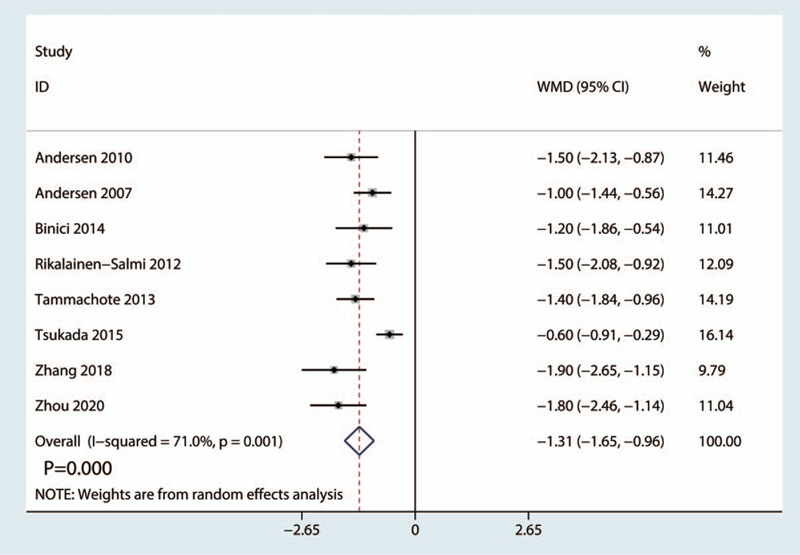
Forest plots of the included studies comparing the length of hospital stay between the 2 groups.

Eight RCTs with 592 patients (LIA = 292, ITM = 300) reported outcomes related to the length of hospital stay. There was high heterogeneity between the included studies (*I*^2^ = 71.0%, *P* = .001, Fig. 3); thus, we adopted a random-effects model to analyze the relevant data. The patients in the LIA group had a significantly shorter length of stay than those in the ITM group (WMD = −1.31, 95% CI: −1.65 to −0.96, *P* = .000; Fig. 3).

### 3.7 Incidence of nausea and vomiting

**Figure 4:**
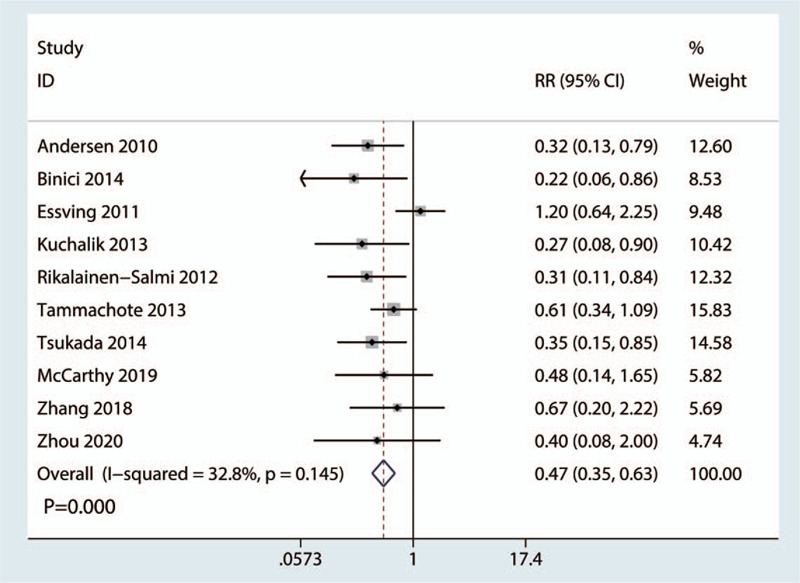
Forest plots of the included studies comparing the occurrence of nausea and vomiting between the 2 groups.

Ten RCTs with 729 patients (LIA = 369, ITM = 360) reported data on the occurrence of nausea and vomiting. There was slight heterogeneity between the included studies (*I*^2^ = 32.8%, *P* = .145, Fig. 4); thus, we adopted a fixed-effects model to analyze the relevant data. The rate of nausea and vomiting was also significantly lower in the LIA group than in the ITM group (RR = 0.46, 95% CI: 0.34–0.63, *P* = .000; Fig. 4).

### 3.8 Incidence of pruritus

**Figure 5:**
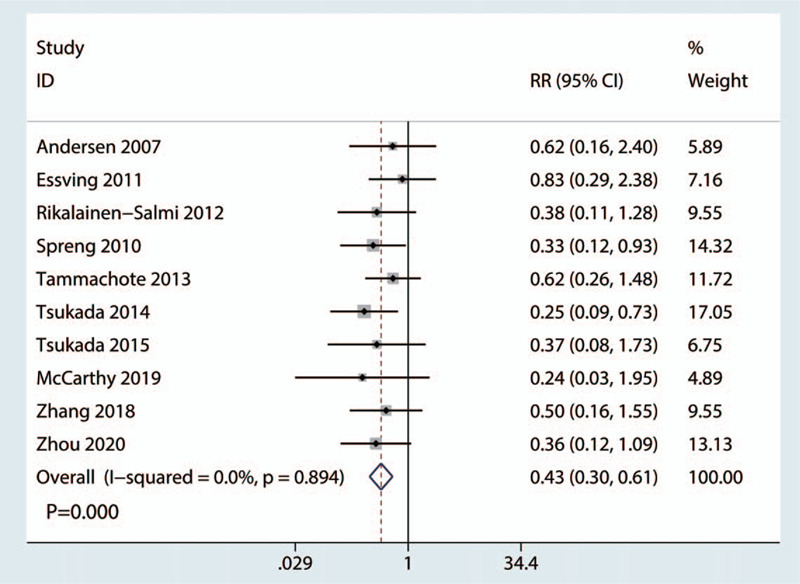
Forest plots of the included studies comparing the occurrence of pruritus between the 2 groups.

A total of 10 studies (760 TKAs, LIA = 398, ITM = 362) were eligible for inclusion in the meta-analysis for the frequency of pruritus. Heterogeneity between the included studies was absent (*I*^2^ = 0.0%, *P* = .894, Fig. 5); thus, we adopted a fixed-effects model to analyze the relevant data. The incidence of pruritus significantly decreased, with a reduction rate of 43% compared with the ITM group (RR = 0.43, 95% CI: 0.30–0.61, *P* = .000; Fig. 5).

### 3.9 Incidence of respiration depression

**Figure 6:**
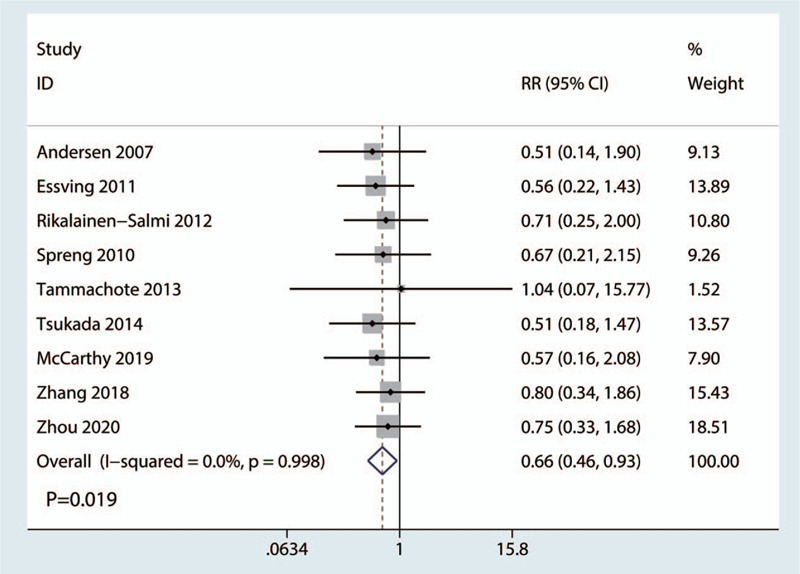
Forest plots of the included studies comparing the occurrence of respiration depression between the 2 groups.

A total of 9 RCTs totaling 720 patients provided data on the occurrence of respiration depression. Heterogeneity between the included studies was absent (*I*^2^ = 0.0%, *P* = .998, Fig. 6); thus, we adopted a fixed-effects model to analyze the relevant data. The frequency of respiration depression was significantly lower in the LIA group than in the ITM group (RR = 0.66, 95% CI: 0.46–0.93, *P* = .019; Fig. 6).

### 3.10 Subgroup analysis, publication bias

**Figure 7:**
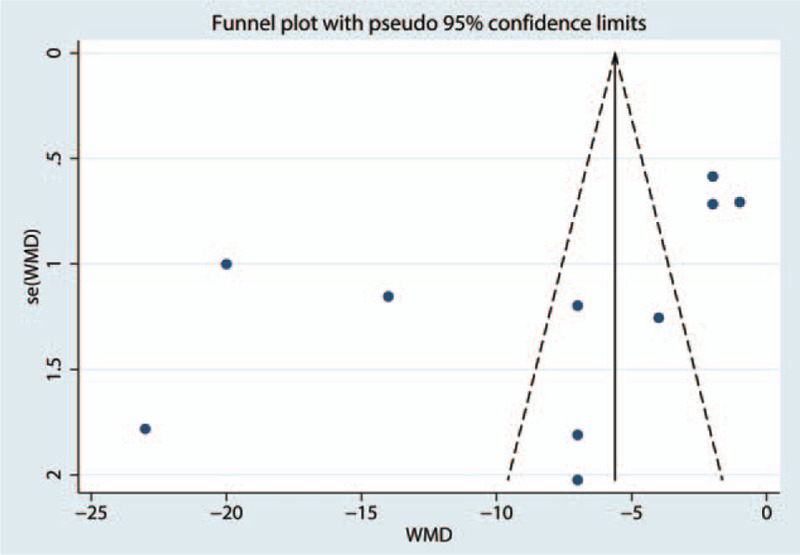
Funnel plot for publication bias of VAS with rest at 6 h.

**Figure 8:**
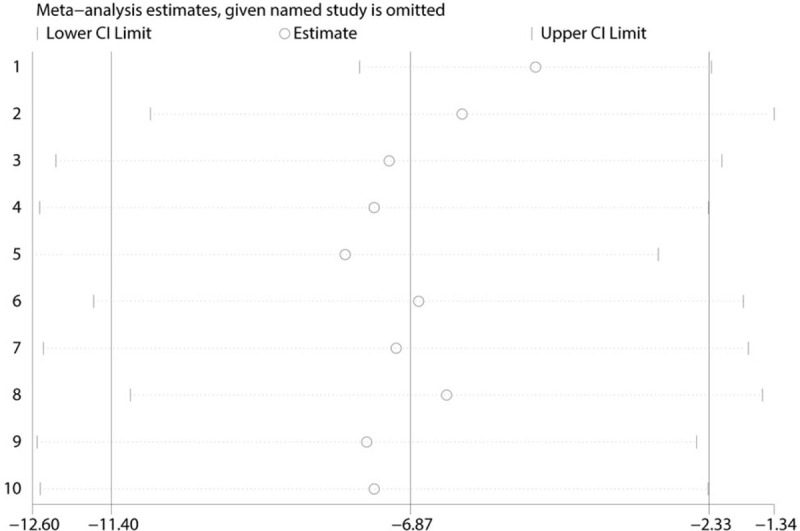
Sensitivity analysis for VAS with rest at 6 h after excluded studies in turn.

Sources of heterogeneity were explored using subgroup analyses and can be seen in Table 3. We found that the effect was independent from the subgroup analysis according to surgery type (TKA or THA). The funnel plot shows that there was no publication bias (Fig. 7) in terms of the VAS score with rest at 6 hours. A sensitivity analysis was performed to address the relative importance of each study by excluding each study in turn from the analysis to evaluate its effect on the pooled WMD and to identify heterogeneous studies. The sensitivity analysis showed that the results remained significant after each study was removed in turn (*P* < .05, Fig. 8).

**Table 3:**
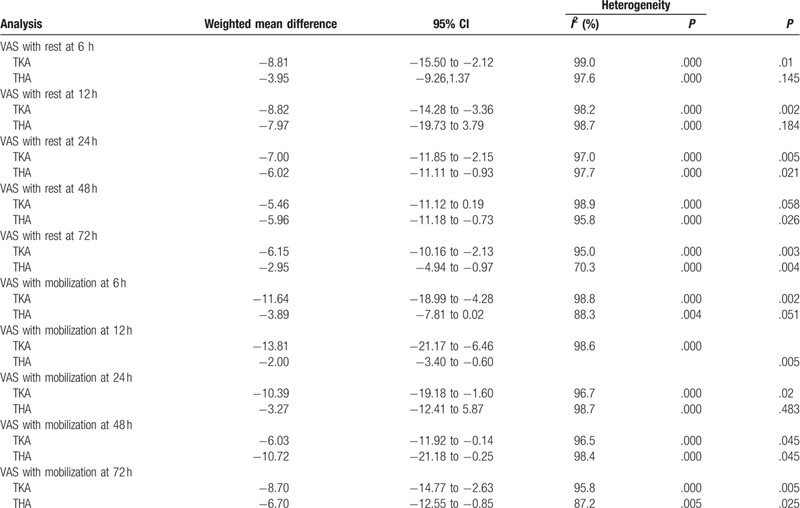
Subgroup analysis for VAS with rest or mobilization at 6, 12, 24, 48, and 72 h according the surgery type.

## 4 Discussion

### 4.1 Main findings

Our review represents the largest meta-analysis conducted to date that included 13 RCTs and 942 patients. The present meta-analysis showed that LIA is better than ITM for TKA and THA patients.

### 4.2 Comparison with other meta-analyses

Two main meta-analyses in this regard have been published,^[23,24]^ but the differences between the meta-analysis in our study and the previous ones should be identified.

First, the previous meta-analyses involved no more than 380 patients. By contrast, our meta-analysis involved 13 trials and 942 patients, which is also the latest and most comprehensive analysis. Further strengths of this meta-analysis include the publication bias and subgroup analysis, which reduce the risk of selection bias.

The pooled results revealed that LIA was superior to ITM in TKA and THA patients. The impacts of pain relief are mainly reflected in the reduction of pain intensity with rest or mobilization for more than 6 hours or even 72 hours. McCarthy et al^[7]^ conducted an RCT and revealed that LIA was superior to ITM for relieving pain at 48 h in TKA patients. Moreover, Jiménez-Almonte et al^[25]^ conducted a systematic review and network meta-analysis and predicted that LIA may be the best treatment measure for TKA and THA patients. However, Adesope et al^[26]^ revealed that local anesthetic wound infiltration had a minimal effect on the pain score.

We next evaluated morphine consumption. Morphine consumption was reduced following TKA and THA when patients received LIA rather than ITM. Askar et al^[27]^ revealed that ITM had an equal pain control efficacy to patient-controlled efficacy. Adesope et al^[26]^ found that local anesthetic wound infiltration had a minimal effect on the pain score but could significantly reduce postoperative opioid consumption. Højer Karlsen et al^[28]^ conducted a review of postoperative pain treatment after THA and showed that LIA provided a morphine-sparing effect of 14.1 (95% CI: 8.0–20.2) mg. We concluded that the above results were similar to the results of our meta-analysis. We found that LIA significantly reduced morphine consumption by approximately 13.52 mg (95% CI: −19.09 to −7.95).

In economic terms, LIA provides a shorter hospitalization time and, last but not least, a better cosmetic outcome than ITM. LIA can also contribute to postoperative pain control as previously documented as a premise for patients’ fast recovery in order to reduce bed-related complications. Moreover, surgeons should pay more attention to morphine-related complications. LIA can significantly reduce morphine-related complications (nausea and vomiting, pruritus, and respiration depression) compared with ITM.

However, this study has the following limitations: the low number of subjects studied and the inadequate sample size within studies; the variable dose of LIA and ITM between studies; the variability of the rehabilitation program between studies, thus potentially affecting the length of hospital stay; and most significantly, the heterogeneity contributing to different doses and intervals among the included studies.

## 5 Conclusion

On the basis of the results of the present analysis, the LIA group had more advantages than ITM in terms of VAS scores during early postoperative periods; moreover, the LIA group may have been subject to lower morphine consumption. Furthermore, participants under LIA did not suffer worse results regarding postoperative complications and VAS scores at 3 months postoperatively. However, further studies with standardized, unbiased methods, and larger sample sizes are still required for deeper analysis.

## Author contributions

**Conceptualization:** Wei-song Qiao.

**Methodology:** Wei-song Qiao.

**Software:** Bao-chang Qi.

**Writing – original draft:** Bao-chang Qi, Jing Yu.

**Writing – review & editing:** Jing Yu.
